# Interleukin-37 Suppresses the Function of Type 2 Follicular Helper T in Allergic Rhinitis

**DOI:** 10.3390/biomedicines13051263

**Published:** 2025-05-21

**Authors:** Xi Luo, Yanhui Wen, Xiangqian Qiu, Lifeng Zhou, Qingxiang Zeng, Wenlong Liu

**Affiliations:** 1 Department of Otolaryngology, Guangzhou Women and Children’s Medical Center, Guangzhou Medical University, Guangzhou 510623, China; nose_law@126.com (X.L.); qiuxiangqian2020@163.com (X.Q.); mapplez@126.com (L.Z.); qingxiangqie@163.com (Q.Z.); 2 Department of Otolaryngology, The First Affiliated Hospital, Jinan University, Guangzhou 510630, China; 13332691157@163.com; 3 Department of Otolaryngology, The Affiliated Dongguan Songshan Lake Central Hospital, Guangdong Medical University, Dongguan 523326, China

**Keywords:** IL-37, allergic rhinitis, Tfh2, IgE, B cells

## Abstract

**Background:** Allergic rhinitis (AR) is triggered by immunoglobulin E (IgE)-mediated immune responses to airborne allergens. Recent studies highlight the pivotal role of T follicular helper 2 (Tfh2) cells in IgE production. Interleukin-37 (IL-37) has emerged as an intrinsic modulator of innate immunity and inflammatory processes. We aimed to investigate the regulatory effect of IL-37 on Tfh2 cells in the pathogenesis of AR. **Methods:** Blood samples were collected from AR patients and controls. The IL-37 levels and the frequency of Tfh2 cells were detected by enzyme-linked immunosorbent assay (ELISA) and flow cytometry, respectively. The isolated Tfh2 cells were cultured or cocultured with naive B cells. The regulatory effects of IL-37 on Tfh2/B cells were assessed using ELISA, quantitative real-time polymerase chain reaction (qRT-PCR). Mouse models of ovalbumin (OVA)-induced AR were established to explore the effect of IL-37 in vivo. **Results:** IL-37 suppressed the production of IL-4 and IL-21 by Tfh2 cells and downregulated C-X-C chemokine receptor type 5 (CXCR5) and B-cell lymphoma 6 protein (Bcl6) mRNA expression while upregulating B lymphocyte-induced maturation protein 1 (Blimp1) and signal transducers and activators of transduction5 (STAT5) mRNA. IL-37 decreased IgE production by B cells significantly, and the addition of anti-IL-18 receptor α alleviated this effect. In mouse models, IL-37 reduced nasal rubbing, sneezing, eosinophil counts, OVA-specific IgE, and Tfh2 proportions. **Conclusions:** IL-37 plays a crucial role in modulating Tfh2 cell responses in AR, suggesting a potential therapeutic target for this condition.

## 1. Introduction

Allergic rhinitis (AR) is distinguished by an immunoglobulin E (IgE)-facilitated immune response triggered by the inhalation of airborne allergens [1]. Epidemiological data indicate that AR affects more than 10% of the global population, with recent studies reporting an escalating prevalence of this condition [2]. Traditionally, T help 2 (Th2) lymphocytes were presumed to orchestrate the class-switch recombination in B lymphocytes, directing their differentiation towards IgE production, via interleukin-4 (IL-4) and interleukin-13 (IL-13) [3]. Nevertheless, recent studies have unveiled a novel T cell subpopulation, group 2 follicular helper T (Tfh2) cells, as the primary mediator in modulating B cell function, thereby supporting antibody responses [4]. This pivotal role of Tfh2 cells is attributed to their strategic localization within B cell follicles and germinal centers, underscoring their central importance in the immunological process [5]. Tfh2 cells have been demonstrated to secrete interleukin-21 (IL-21) and identified as the main source of IL-4 cytokine generation within secondary lymphoid organs [6].

Several studies have revealed the expansion of Tfh2 cells and the synthesis of IgE in the presence of allergens [7,8]. In human subjects allergic to house dust mites (HDM), an increase in the abundance of Tfh2 cells and a decrease in the proportion of Tfr (T follicular regulatory) cells have been observed [9,10]. Notably, Tfh2 cells in HDM-allergic patients demonstrated an augmented ability to stimulate IgE production compared with those from healthy individuals.

Interleukin-37 (IL-37) is a pivotal regulator of both innate and adaptive immunity, modulating inflammatory processes via the IL-18Rα/IL-1R8 receptor complex [11,12]. The IL-1R8 (SIGIRR) is recognized as the primary coreceptor for IL-37’s anti-inflammatory signaling [13]. Dysregulated expression patterns of IL-37 have been documented in a spectrum of inflammatory disorders [14]. Our prior research has delineated a significant decrease in the concentrations of IL-37 in both serum and nasal lavage samples obtained from children with AR [15]. Moreover, Li reported increased expression of IL-1R8 and a possible immunomodulatory role of its ligand IL-37 in AR [16]. In vitro experimental models have clarified that IL-37 exerts an inhibitory influence on the differentiation of naïve CD4+ T cells towards the Th2 cellular subsets [17]. Paralleling these in vitro observations, in vivo studies utilizing HDM-induced AR murine models have demonstrated that topical administration of IL-37 mitigates allergic manifestations and modulates the level of cytokines associated with Th2 and Th17 pathways within the nasal mucosa [18]. However, the mechanisms linking IL-37 to Tfh2-mediated IgE production remain unexplored.

The current investigation was aimed at exploring the impact of IL-37 on the modulation of Tfh2 cells in AR through both in vivo and in vitro studies. Our study may provide a new potential treatment target for AR.

## 2. Methods

### 2.1. Patient Recruitment

Sixteen HDM-sensitive AR patients were enrolled alongside twenty asymptomatic controls, following approval from the local ethics committee (No. 261A01) and with informed consent secured from all participants. AR was ascertained by persistent symptoms for over one year and confirmed by positive skin prick test or specific IgE reactivity to *Dermatophagoides pteronyssinus* and/or *Dermatophagoides farina*, as previously delineated in the literature [19]. Exclusion criteria were stringently applied, excluding individuals with immunological disorders, recent corticosteroid use (4 weeks), smoking history, or concurrent allergic conditions such as asthma or atopic dermatitis. The control group was selected for the absence of nasal symptoms and negative allergen testing. To assess the intensity of symptoms, a total nasal symptom score (TNSS) was utilized, which scales from 0 (representing no distress) to 3 (indicating severe distress) for individual symptoms, including itchiness, sneezing, nasal congestion, and rhinorrhea.

### 2.2. Blood Samples Preparation

Venous blood samples were collected and subjected to centrifugation at a relative centrifugal force of 1000× *g* for a duration of 15 min at a controlled temperature of 4 °C. These serum samples were preserved at −80 °C and utilized for further analysis.

### 2.3. Enzyme-Linked Immunosorbent Assay (ELISA)

The concentrations of cytokines (IL-4, IL-21, IL-37, immunoglobulin G (IgG), immunoglobulin M (IgM), immunoglobulin A (IgA), IgE, CXC motif chemokine ligand 13 (CXCL13)) were determined using ELISA kits provided by Thermo Fisher Scientific (Waltham, MA, USA).

### 2.4. Quantitative Real-Time Polymerase Chain Reaction (qRT-PCR)

Total RNA was isolated from serum samples using the RNeasy Mini Kit. Following this, 1 microgram of RNA was reverse transcribed into cDNA by the Qiagen (Hilden, Germany) cDNA synthesis kit. The relative abundance of the target gene was calculated using the formula 2−ΔΔCt and normalized against an endogenous reference gene. The primer pairs were used as follows: C-X-C chemokine receptor type 5 (CXCR5): forward, AACGTCCTGGTGCTGGTGA, reverse, CACGGCAAAGGGCAAGA; B-cell lymphoma 6 protein (Bcl6): forward, CCAGCAAAGAAGAAGAGAGACC, reverse, CTGTGGACTAACCAGACCCTTC; B lymphocyte-induced maturation protein 1 (Blimp1): forward, AGCTTTCATCCCCTCGTACAAC, reverse, CGCTCAGGCCATTACAATTCAT; signal transducers and activators of transduction5 (STAT5): forward, GAAAGCATG AAAGGGTTGGAG, reverse, AGCAGCAACCAGAGGACTTAC; and activation-induced cytidine deaminase (AID): forward, AAAATGTCCGCTGGGCTAAG, reverse, TCGTGGTTTTCTTTGAAGGTCAT; IL-18Rα: forward, TGA CTC CAG AAG GCAAAT GGC, reverse, AAA GAG ATT TAT CGG CCT TCC, β-actin: forward, TCCTGTGGCATCCACGAAACT, reverse, GAAGCATTTGCGGTGGACGAT.

### 2.5. Flow Cytometry for Tfh2 Cells

Peripheral blood mononuclear cells (PBMCs) were purified utilizing Ficoll–Paque PLUS (Fenghao, China), according to previously reported protocols [20]. To mitigate nonspecific binding, cells were pre-incubated with 5% fetal bovine serum (FBS, Fenghao, China) for 10 min on ice, prior to staining with a fixable viability dye (Fenghao, China) to exclude nonviable cells. Surface antigens were then labeled with primary antibodies (mouse monoclonal antibodies against CD4, PD1, CD25, CXCR3, CCR6, CD3, CD19, CD27, IgD, and human recombinant antibody against CXCR5 for 30 min at 25 °C. All the antibodies used in the staining were purchased from Biolegend (San Diego, CA, USA). Flow cytometric analysis was performed utilizing the BD LSRFortessa™ X-20 instrument from BD Biosciences (Franklin Lakes, NJ, USA) coupled with FlowJo 10.0 software. CXCR3^−^CCR6^−^CD4^+^CXCR5^+^PD-1^+^ were defined as Tfh2 cells as described previously [21] (Figure S1).

### 2.6. Cell Sorting

Fluorescence-labeled PBMCs were subjected to precise sorting of peripheral B cell and T cell subsets utilizing a BD FACSAria II cell sorter (BD Biosciences). For the isolation of naive B cells, the gating strategy was designed to select CD3^−^CD19^+^CD27^−^IgD^+^ cells. Tfh2 cells were identified and sorted based on the phenotype CXCR3^−^CCR6^−^CD4^+^CXCR5^+^PD-1^+^ cells. Post-sorting, the purity of all sorted cell populations exceeded 95%, ensuring the integrity of downstream analyses.

For stimulation experiment, sorted Tfh2 cells (5 × 10^4^ cells) were seeded for a duration of 5 days in RPMI-1640 medium (Fenghao, China) supplemented with 10% heat-inactivated FBS (Fenghao, China) and 1% antibiotics (Fenghao, China) in the presence of 20 μg/mL Der p 1 (R&D system, Minneapolis, MN, USA). The IL-37 (10–100 ng/mL, R&D system, USA) and anti-IL-18Rα (100 ng/mL, clone BG/218901, rat IgG2a; Shanghai C-reagent Biotechnology, Shanghai, China) were added to different groups.

For Tfh2–B-cell coculture, sorted Tfh2 cells (5 × 10^4^ cells) were cocultured with naive B cells (2.5 × 10^4^ cells) for 8 days in the same medium as described above in the presence of staphylococcal enterotoxin B (1 μg/mL; Toxin Technology, Sarasota, FL, USA) in U-bottom 96-well plates. The IL-37 (10–100 ng/mL) and anti-IL-18Rα (100 ng/mL) were added for blocking experiments.

The concentrations of IL-37 were selected based on dose–response pilot experiments and prior studies demonstrating efficacy in human Tfh cell/B cell systems [22].

### 2.7. Mouse Model

Eight-week-old female BALB/c mice were randomly allocated to four groups, with six animals per group. For inducing an ovalbumin (OVA)-sensitized AR model, mice received intraperitoneal injections of OVA (1 mg/mL, Invivogen, San Diego, CA, USA) and aluminum hydroxide (20 mg/mL, Sigma-Aldrich, St. Louis, MO, USA) in saline on days 1, 3, 5, 7, 9, 11, and 13. From days 20 to 30, mice were intranasally challenged with OVA (60 mg/mL). For the IL-37 and/or anti-IL-18Rα interventions, 0.02 mg/mL IL-37 (R&D Systems, Minneapolis, MN, USA) or 0.02 mg/mL anti-IL-18Rα (Shanghai C-reagent Biotechnology, Shanghai, China) was administered intraperitoneally 30 min prior to each OVA challenge and continued for five days (days 26 to 30). Control mice were administered saline instead of OVA. The times of sneezing and nasal rubbing were recorded by two blinded observers over a 10 min period after the final challenge on day 30. Our animal studies obtained approval from the Guangdong Medical Laboratory Animal Center (reference number: C202306-31). Our research adhered to the Guidelines for Ethical Review of Chinese Experimental Animal Welfare.

### 2.8. Histologic Analysis

Within a 24 h interval subsequent to the final experimental challenge on the thirtieth day, all murine subjects were sacrificed. Segments of the nasal mucosa were excised, subsequently fixed in a 4% paraformaldehyde solution. These tissues underwent a series of histological processes, including dehydration, paraffin embedding, and sectioning into 4–5 μm thin slices. The prepared sections underwent hematoxylin and eosin (H&E) staining to allow for detailed examination under a microscope. The quantification of eosinophils was performed on four randomly selected high-power fields (400× magnification) to assess the inflammatory cellular infiltration.

### 2.9. Statistical Analysis

The dataset was statistically analyzed by SPSS 20.0. The results were presented as mean values accompanied by the standard error of the mean (SEM). Data distribution was assessed using Shapiro–Wilk tests. Nonparametric tests (Mann–Whitney U and Spearman’s correlation) were applied to between-group comparisons, while parametric tests (Student’s *t*-test and ANOVA) were used only for normally distributed within-group data (e.g., IL-37 dose–response). Correlation analysis was performed using Spearman’s rank correlation coefficient. Statistical significance was determined at a *p*-value less than 0.05.

## 3. Results

### 3.1. Serum Protein Concentration of IL-37 and Its Correlation with the Frequencies of Tfh2 in AR

**Figure 1:**
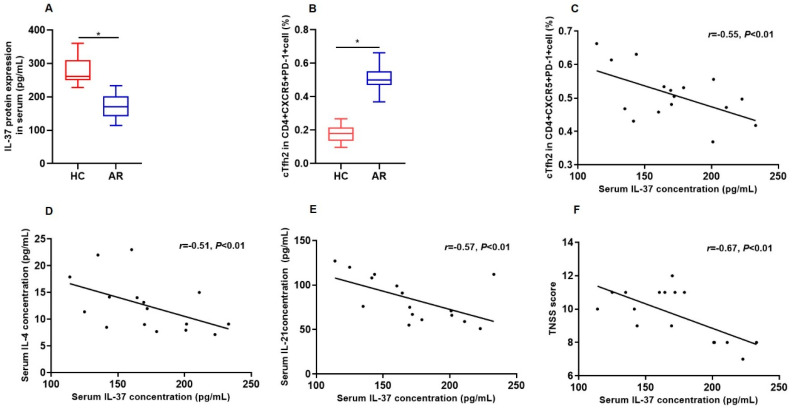
IL-37 protein expression in serum and correlation with the percentage of peripheral blood Tfh2 in AR: (**A**) IL-37 protein expression in serum in AR (*n* = 16) and controls (*n* = 20) detected by enzyme-linked immunosorbent assay (ELISA). (**B**) The percentage of peripheral blood Tfh2 between AR (*n* = 16) and controls (*n* = 20) detected by flow cytometry. (**C**–**F**) The correlation between IL-37 protein expression in serum and the peripheral frequencies of Tfh2, serum IL-4 protein expression, serum IL-21 protein expression, and TNSS score. HC, healthy control; AR, allergic rhinitis; TNSS, total nasal symptom score. * Compared with AR group, *p* < 0.05.

The characteristics of the AR patients and control subjects are detailed in Table 1. The serum levels of IL-37 protein in AR were significantly lower compared with controls (*p* < 0.001) (Figure 1A). Additionally, the frequencies of circulating Tfh2 cells were notably lower in the control group compared with AR patients (*p* < 0.001) (Figure 1B). The serum IL-37 expression in AR patients was negatively correlated with the proportions of circulating Tfh2 cells, serum IL-4 and IL-21 protein levels, and the TNSS in AR patients (Figure 1C–F).

### 3.2. The Direct Effect of IL-37 on Tfh2 Cells

**Figure 2:**
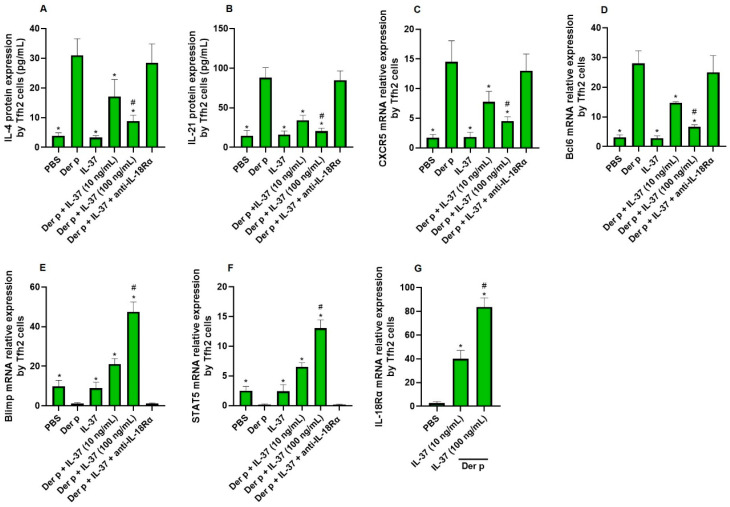
The effect of IL-37 on Tfh2 cells: (**A**,**B**) The expression of IL-4 and IL-21 protein by Tfh2 cells regulated by IL-37 detected by ELISA (*n* = 6). (**C**–**G**) The mRNA expression of CXCR5, Bcl6, Blimp, STAT5 and IL-18Rα by Tfh2 cells regulated by IL-37 (*n* = 6). For (**A**–**F**): *, Compared with Der p group, *p* < 0.05. ^#^, Compared with Der p+ IL-37 (100 ng/mL) group, *p* < 0.05. (**G**): *, Compared with PBS group, *p* < 0.05. ^#^, Compared with IL-37 (10 ng/mL) group, *p* < 0.05.

Our findings indicate that Der p stimulates the production of IL-4 and IL-21 proteins by Tfh2 cells compared with phosphate-buffered saline (PBS). However, these effects were significantly suppressed by IL-37 in a dose-dependent manner (Figure 2A,B). Moreover, the CXCR5 and Bcl6 mRNA expression by Tfh2 cells was also downregulated by IL-37 (Figure 2C,D). On the contrary, the Blimp1 and STAT5 mRNA expression by Tfh2 cells was upregulated by IL-37 (Figure 2E,F). When anti-IL-18Rα was added to the above system, the inhibitory effect of IL-37 was significantly alleviated. Our data also showed that IL-18Rα mRNA expression by Tfh2 cells was upregulated significantly by IL-37 (Figure 2G).

### 3.3. The Interactions Between Tfh2 and B Cells Regulated by IL-37

**Figure 3:**
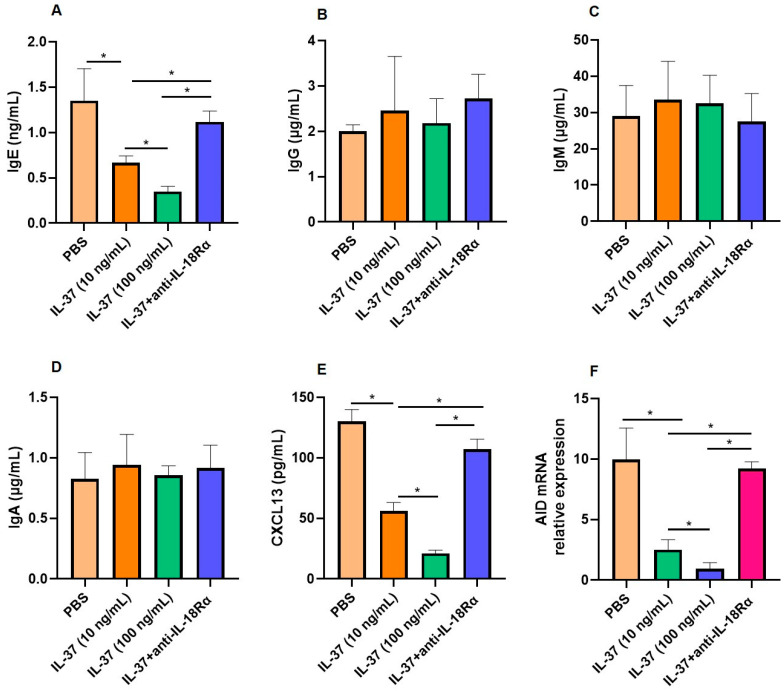
Inhibitory effect of IL-37 in Tfh2–B-cell coculture system: (**A**–**E**) The protein expression of IgE, IgG, IgM, IgA, and CXCL13 protein by Tfh2–B-cell coculture system regulated by IL-37 detected by ELISA (*n* = 6). (**F**) The AID mRNA relative expression by Tfh2–B-cell coculture system regulated by IL-37 was detected by PCR (*n* = 6). * Compared between groups, *p* < 0.05.

We cocultured Tfh2 and B cells to confirm the modulatory role of IL-37 on these cells. IL-37 decreased IgE production by B cells significantly, and the addition of anti-IL-18Rα alleviated this effect (Figure 3A). Interestingly, the production of IgG, IgM, and IgA by B cells was not affected by IL-37 (Figure 3B–D). We also found that IL-37 downregulated the levels of CXCL13 and AID, and the addition of anti-IL-18Rα alleviated these effects (Figure 3E,F).

### 3.4. IL-37 Suppresses Tfh2 Response in an Allergic Mouse Model

**Figure 4:**
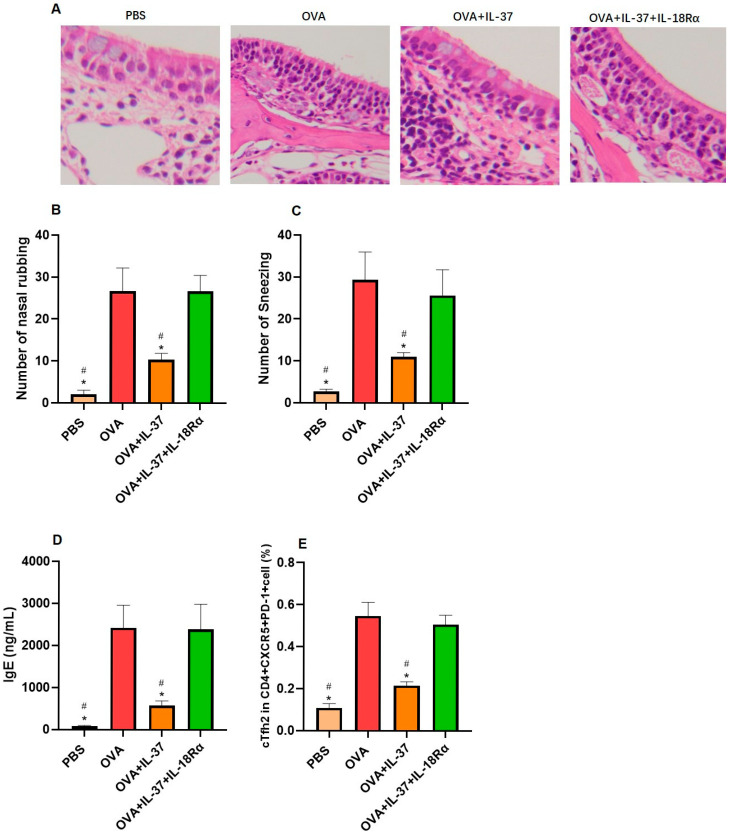
IL-37 inhibited peripheral blood Tfh2 proliferation in the mouse model: (**A**) HE staining of nasal section among different groups (×400). (**B**,**C**) The allergic symptoms of mice in different groups. (**D**) The OVA-specific IgE expression in serum among different groups. (**E**) The frequencies of Tfh2 among different groups. * Compared with groups, *p* < 0.05. ^#^ Compared with OVA+IL-37+ anti- IL-18Rα group, *p* < 0.05.

In IL-37-treated mice, the eosinophil counts, the number of nasal rubbing and sneezing, the OVA-specific IgE concentration, and the proportion of Tfh2 were significantly attenuated compared with OVA-sensitized mice (Figure 4). However, anti-IL-18Rα treatment alleviated the above effect of IL-37 (Figure 4).

## 4. Discussion

In recent years, the role and mechanisms of Tfh2 cells in allergic diseases have garnered substantial attention [6]. Their primary function in allergy is to facilitate the expression of IgE in germinal centers (GCs) of B cells through the expression of IL-4, thereby contributing to the initiation and progression of allergic diseases [6]. However, to date, the regulatory mechanisms underlying Tfh2 cells remain incompletely elucidated. In this study, we elucidate the negative role of IL-37 on the proliferation and function of Tfh2 cells in allergic rhinitis for the first time.

Here, we explored the role of IL-37 in regulating Tfh2 cells in AR. Consistent with previous studies, AR patients exhibited significantly lower serum IL-37 expression and higher Tfh2 cell frequencies than controls [9,10,15,16,23]. Moreover, we confirmed that IL-37 expression was negatively correlated with Tfh2 cell proportions, IL-4/IL-21 levels, and AR severity. Therefore, we further explored the direct effect of IL-37 on Tfh2 cells. As expected, IL-37 inhibited Der p-induced Tfh2 cell expansion and function, evidenced by reduced IL-4/IL-21 expression and altered CXCR5/Bcl6/Blimp1/STAT5 mRNA levels. Of which, CXCR5 and Bcl6 are positive factors that can initiate the differentiation of Tfh cells, while blimp1 and STAT5 are negative factors that inhibit the differentiation of Tfh cells [24]. Our findings offer primary, direct evidence supporting the suppressive regulatory function of IL-37 on Tfh2 cells in the pathogenesis of AR. Similarly, Liu’s study also proved that IL-37 directly inhibited the proliferation and cytokine production by Tfh cells in Myasthenia Gravis [22]. They also reported that IL-37 combines with cell surface IL-18Ra and the orphan decoy IL-1 family receptor 8 (SIGIRR) to exert its effect [22]. Unlike Liu et al., who implicated SIGIRR (IL-1R8) in IL-37’s effects, our data specifically highlight IL-18Rα as the dominant receptor mediating Tfh2 suppression in AR. This receptor-specific mechanism was validated through anti-IL-18Rα blocking experiments, revealing a divergent signaling axis compared with the SIGIRR/STAT3 pathway reported in Myasthenia Gravis.

Tfh2 cells facilitate B cell differentiation and proliferation within GCs, thereby contributing to the establishment and preservation of GCs as well as the generation of high-affinity, class-switched antibodies [4]. We demonstrated that IL-37 suppressed IgE secretion by B cells cocultured with Tfh2 cells, while the production of IgG, IgM, and IgA was not affected. The possible reason lies in the addition of Der p in the coculture system, which results in a state where class-switch recombination is predominantly IgE-based. Moreover, we found that IL-37 downregulated the expression of CXCL13 and AID in a dose-dependent manner. Of which, CXCL13 plays a vital role as a chemokine in guiding B cells and Tfh2 cells to their destinations [4]. The enzyme activation-induced cytidine deaminase (AID) is crucial for activating B cells and facilitating antibody class switching [25]. These results suggested that IL-37 primarily inhibits IgE production by regulating the expression of CXCL13 and activation of AID. We also confirmed our result by an allergic mouse model. IL-37 treatment reduced allergic symptoms, eosinophilia, and Tfh2 cell proportions, while these effects can be reversed by anti-IL-18Rα. While BALB/c mice exhibit inherent type 2 polarization, our findings highlight IL-37’s capacity to suppress Tfh2-driven allergic responses even within a Th2-skewed milieu. This suggests therapeutic potential across diverse immunological contexts, though validation in other models remains warranted.

Two distinct mechanisms of IL-37 in modulating immune suppression were posited, including intracellular translocation of IL-37 to the nucleus and extracellular combination with IL-18Ra and SIGIRR [26,27,28,29]. Our results confirm the crucial role of the surface receptor IL-18Rα in IL-37-mediated suppression of Tfh2 cells. This conclusion is primarily based on the observation that antagonism of this receptor almost completely abrogates the effect of IL-37.

This study has some limitations. First, our sample size is relatively small. Future investigations with larger cohorts and diverse populations are warranted to further validate the regulatory role of IL-37 in Tfh2-mediated allergic responses. Secondly, while our findings emphasize the importance of IL-18Rα in IL-37-mediated suppression of Tfh2 cells, the role of SIGIRR (IL-1R8), another receptor implicated in IL-37 signaling, was not investigated. Future studies should explore whether SIGIRR contributes synergistically or independently to IL-37’s immunomodulatory effects in allergic rhinitis. Thirdly, our study focused on acute-phase responses to IL-37, and the long-term therapeutic implications, including effects on immunological memory or tolerance induction, remain unexplored. Future investigations employing extended treatment regimens and rechallenge protocols will be necessary to evaluate whether IL-37 can induce durable remission or alter the natural history of allergic inflammation.

In summary, our research highlights the pivotal function of the IL-37–IL-18Rα signaling pathway in modulating the interactions between Tfh and B cells in AR. Our investigation could offer a fresh potential therapeutic target for AR.

## Figures and Tables

**Table 1:** Characteristic of patients with allergic rhinitis (AR) and healthy controls.

Groups	AR	Control
Number	16	20
Sex (Male:Female)	8:8	11:9
Age (years)	21.2 ± 2.9	22.3 ± 3.6
Duration of symptoms, (years)	2.1 ± 1.4	-
Serum sIgE level to Der p (IU/mL) Serum sIgE level to Der f (IU/mL)	21.8 (6.3–599.4) 30.5 (2.4–462.8)	- -

## Data Availability

The data can be obtained from the corresponding author.

## Supplementary Materials

The following supporting information can be downloaded at: https://www.mdpi.com/article/10.3390/biomedicines13051263/s1, Figure S1: The gating strategy of Tfh2 cells.
