# Supplementary material for: Interleukin-37 Suppresses the Function of Type 2 Follicular Helper T in Allergic Rhinitis
Source: Biomedicines. 2025 May 21;13(5):1263. doi: 10.3390/biomedicines13051263 (PMC12108951; doi:10.3390/biomedicines13051263)
Supplement: Supplementary file 1 [file biomedicines-13-01263-s001.zip › biomedicines-3622350-supplementary.pdf]

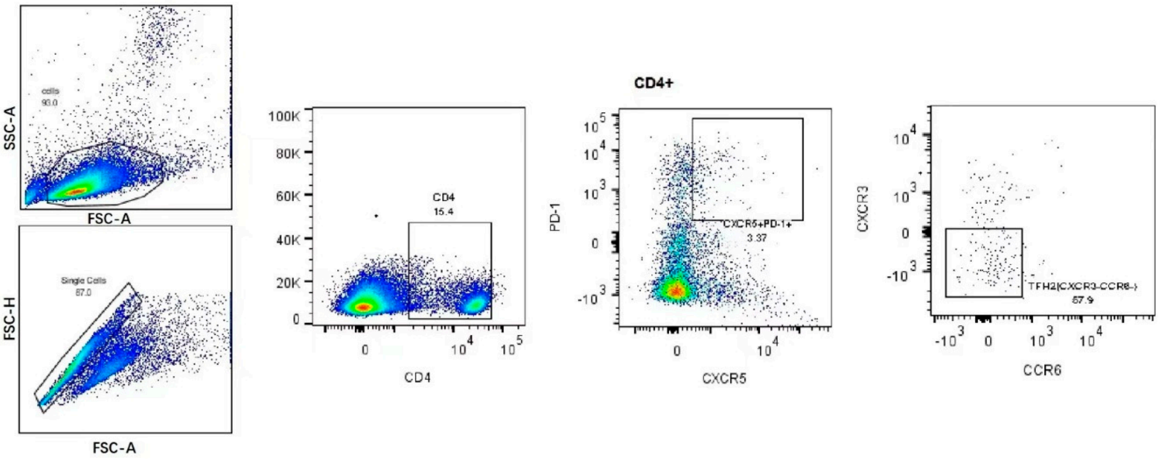

Figure S1. Gating strategy of peripheral blood Tfh2 cells. $CXCR3^{-}CCR6^{-}CD4^{+}CXCR5^{+}PD\text{-}1^{+}$ were defined as Tfh2 cells.
